# Exploring an Efficient Evolutionary Game Model for the Government–Enterprise–Public during the Double Carbon Policy in China

**DOI:** 10.3390/ijerph19084607

**Published:** 2022-04-11

**Authors:** Bilin Zou, Chunhua Ju, Fuguang Bao, Ye Lai, Chonghuan Xu, Yiwen Zhu

**Affiliations:** 1 School of Management and E-Business, Zhejiang Gongshang University, Hangzhou 310018, China; bilinzou@126.com (B.Z.); jch@zjgsu.edu.cn (C.J.); baofuguang@126.com (F.B.); 2 Contemporary Business and Trade Research Center, Zhejiang Gongshang University, Hangzhou 310018, China; 3 Academy of Zhejiang Culture Industry Innovation & Development, Zhejiang Gongshang University, Hangzhou 310018, China; 4 School of Electronic and Communication Engineering, Sun Yat-sen University, Shenzhen 518107, China; laiy28@126.com; 5 School of Business Administration, Zhejiang Gongshang University, Hangzhou 310018, China; 6 School of Chinese Language and Literature, Zhengzhou University, Zhenzhou 450000, China; zyw19846751865@163.com

**Keywords:** CPAN goals, carbon labelling system, low-carbon technology R&D, act of government, evolutionary game

## Abstract

The realization of China’s “double carbon” goal is of great significance to the world environment and China’s economy and society. Through the establishment of the “government–enterprise–public” evolutionary game model, this paper explores the interaction between government policy guidance, low-carbon technology R&D behavior of enterprises, and public purchase of carbon label products, as well as the micro-driving path, aiming to provide suggestions for the implementation of the “double carbon” policy and carbon label system in China. The results show that the choice of government, enterprises, and public strategies is closely related to their own costs and benefits. Public sentiment can effectively urge the government to actively fulfill its responsibilities. Effective government policy guidance plays a key role in low-carbon technology R&D behavior of enterprises. There is an interaction between low-carbon technology R&D behavior of enterprises and public purchase of carbon label products.

## 1. Introduction

Since the goal of CPAN (“carbon peak” and “carbon neutralization”) was proposed in the *Paris Agreement*, which was signed on 12 December 2015, China has been always committed to achieving it and has formulated a series of relevant policies [1,2]. The ultimate realization of the CPAN goal largely depends on the wide application of low-carbon technology in both production and life [3,4]. There are many factors that can influence the innovation of low-carbon technology, among which the most important are the guidance of government policies, enterprises’ R&D investment in low-carbon technology, and public purchase of carbon label products. The guidance of government policy is the driving force for enterprises to innovate low-carbon technology, and the R&D investment of enterprises directly affects the innovation of low-carbon technology. The continuous purchase of carbon label products by consumers can also encourage enterprises to conduct research and development of low-carbon technologies. In recent years, due to the frequent occurrence of environmental problems, the government’s environmental supervision task is heavy, and the government’s supervision and guidance ability is limited. In order to achieve the goals of CPAN and promote the R&D of low-carbon technology and the establishment of a carbon labeling system, enterprises and the public also need to participate. But at present, the implementation of relevant policies in China lacks binding force, which is dominated by the government and supplemented by voluntary participation of enterprises and the public. How to stimulate enterprises’ low-carbon technology R&D and people’s purchase of carbon label products through government’s guidance, and how to build a good market environment for enterprises and the public to interact with each other, and ultimately promote the realization of CPAN goals are still worthy of discussion.

Based on this, in order to explore the interaction and micro-driving path between governmental policy guidance, enterprises’ low-carbon technology R&D behavior and the public’s purchase of carbon label products, this paper first takes the government and enterprises as the main subjects. The influencing factors of public opinion are introduced, and an evolutionary game model of both the government and enterprises considering public opinion is established to explore the interaction between government and enterprise behavior and the influence of public opinion on government and enterprise behavior. Secondly, taking enterprises and the public as the main body, this paper introduces the intensity factor of government policy implementation, establishes an evolutionary game model of enterprises and the public under the guidance of the government, and explores the interaction between enterprises and public behavior and the influence of policy implementation strength on enterprises and public behavior. Finally, an evolutionary game model of the government, enterprises and the public is established to explore the interaction among government policy guidance, enterprises’ low-carbon technology R&D behavior and public’s purchase of public carbon label products. By controlling different parameters in the form of numerical simulation, the micro-driving mechanism and path of different subjects are explored, and the influence of different parameters on the behavior choice of the government, enterprises and the public is obtained, so as to provide optimization suggestions for the smooth implementation of China’ s CPAN policy and carbon labeling system.

## 2. Literature Review

At present, whether government policies can induce low-carbon technology R&D is a hot topic in academia. Fan [5] found that environmental regulation, foreign direct investment, and the technological level in government fiscal expenditure have a positive impact on regional low-carbon technology R&D behavior. Zhang [6] divided low-carbon technology R&D into environmental-induced R&D and production-oriented R&D, and found that green credit regulatory policies can significantly improve the growth of green total factor productivity (GTFP), indicating that environmental-induced R&D was the driving force of GTFP. And production-oriented R&D can significantly improve the input-output total factor productivity. Wang [7] collected panel data of 11 provinces along the Yangtze River Economic Belt from 2008 to 2017, and used slacks-based measures (SBM) -data envelopment analysis (DEA) model and panel Tobit model to show that government R&D subsidies and environmental regulations are conducive to improving the R&D efficiency of low-carbon technologies in the Yangtze River Economic Belt. Song [8] collected data from 30 regions in China from 2009 to 2017, and proposed a panel model to analyze the data. There was a U-shaped relationship between environmental policy regulation and green product innovation. At present, the level of supervision by the Chinese government needs to be further improved. The above studies have confirmed that government policies can induce macro low-carbon technology R&D behavior, but do not pay attention to the impact of government policies on corporate R&D behavior. Nie [9] believed that imposing an emission tax can effectively promote enterprises’ low-carbon technology R&D behavior. Xu [10] collected data from 223 listed companies in China from 2015 to 2018, and used multiple regression analysis to prove that environmental, social and governance performance can increase the number of green invention patents. Bai [11] studied the samples of 527 listed companies and found that government R&D subsidies increased the trend and performance of low-carbon technology R&D of energy-intensive enterprises by 107.3% and 54.1%, respectively. Heterogeneity analysis proved that government R&D subsidies had a greater impact on state-owned enterprises and small and medium-size enterprises. As the main implementation object of CPAN policy, enterprises’ low-carbon technology R&D behavior is of great significance to the realization of CPAN goals. The above studies show that government policies can promote enterprises’ low-carbon technology R&D behavior in different degrees. Lin [12] proved that green innovation strategy has a positive impact on brand value, and this effect is more obvious for enterprises with high R&D intensity. Grisales [13] proved that increasing the investment level of low-carbon technology R&D has a positive regulatory effect on corporate financial performance. Thus, in addition to the guidance of government policies, the active participation of market and consumers is also a key factor in promoting low-carbon technology R&D.

Carbon label refers to the form of label attachment to reflect the carbon emissions generated in the life cycle of products or services [14]. The carbon labeling system was proposed by the United Kingdom in 2006, and has been successfully implemented in the United States, France, South Korea, Japan and other countries [15]. Although the carbon labeling system has not been fully implemented in China, scholars have conducted in-depth research on the development, production and promotion of carbon label products. For the purchase behavior of public carbon label products, Zhao [16] took Chengdu, China, as an example. The results show that people’s awareness of carbon label products is generally low, and perceived benefits significantly affect people’s willingness to buy carbon label products. Mostafa [17,18,19] considered that the purchase of carbon label products is a complex decision-making process involving gender, age, education and income level. For the R&D behavior of enterprise carbon label products, Liu [20] proved that one of the main motivations for enterprises to attach carbon labels to products is marketing, and carbon label products can enhance people’s consumption behavior. Gadema [21,22] proved that most enterprises are profit-driven, and they may less likely to choose carbon labels considering the impact of costs and benefits such as government policies and market risks. However, the above scholars only considered the production and consumption of carbon label products from a single perspective of the public or enterprises, and did not consider the interaction among government policy guidance, enterprise low-carbon technology R&D behavior and public carbon label product purchase behavior in the market environment. In fact, in recent years, the interaction between multi-stakeholders in the related research of carbon label has gradually attracted the attention of the academic community [23]. The game model of multi-agent relationship analysis can provide a more specific research framework for exploring the interaction and micro-driving path among government policy guidance, low-carbon technology R&D behavior of enterprises and public’s purchase of public carbon label products in the market environment. The existing research uses the game method to extensively discuss the strategic choices and influencing factors of all parties under the carbon labeling system. Zhao [24,25] studied the impact of product prices and price subsidies on consumers’ purchase of carbon label products, but this study only considered the role of government on consumers and the interaction between consumers. Han [26] studied the impact of carbon label products on the strategic choices of enterprises and consumers in the market, but did not consider the government as the main body of the game. Liu [27,28] studied enterprises’ low-carbon technology R&D behavior and carbon label production behavior under government supervision, but did not consider the impact of the public. However, in real life, government policy guidance, low-carbon technology R&D behavior of enterprises and public’s purchase of carbon label products often influence each other. Chen [29] established a tripartite evolutionary game model considering the interaction among the government, manufacturers and the public under carbon taxes and subsidies. Xu [30] explored the impact of government and the public on green technology innovation behavior of enterprises through evolutionary game method. However, in the above literature, the public participation behavior is limited to public supervision. The influence of the market value and potential value of carbon label products provided by enterprises on enterprises’ low-carbon technology R&D behavior is not considered, nor is the influence of the value of carbon label products provided to the public on the public’s purchase of carbon label products.

In summary, the existing literature has explored the driving path of government policy guidance, low-carbon technology R&D behavior of enterprises and public purchase of carbon label products at the macro and micro levels, respectively, but there is a lack of research on the interaction between the three and the micro driving path. Based on this, this paper establishes a game model among the government, enterprises and the public through the evolutionary game method, and studies how the government promotes enterprises to conduct low-carbon technology R&D and the public to purchase carbon label products under the CPAN target and carbon labeling system, and how to build a good market environment for carbon label products through the interaction between enterprises and the public [31,32,33,34,35,36,37].

## 3. The Evolutionary Game between Multi-Agents under the Background of CPAN Policy and Carbon Labeling System

**Figure 1:**
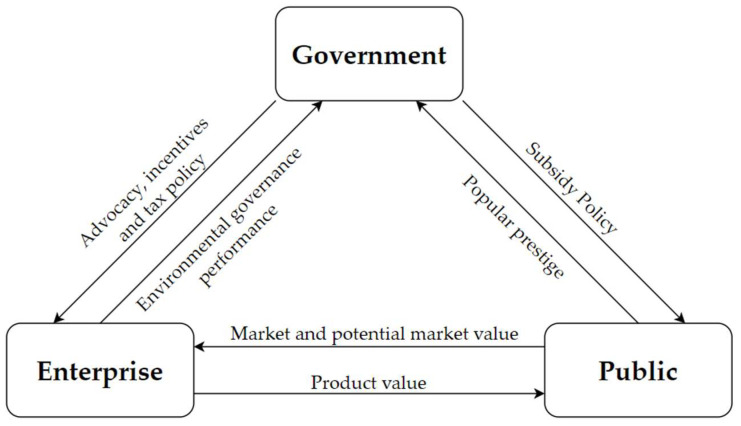
Multi-agent driving mechanism under the background of CPAN policy and carbon labelling system.

As shown in Figure 1, this paper expounds the driving mechanism among the government, enterprises, and the public under the background of the implementation of the CPAN policy and the carbon label system from the following three aspects. For the government and enterprises, the government’s publicity, incentives, and tax policies can promote enterprises’ low-carbon technology R&D behavior, and enterprises’ low-carbon technology R&D behavior can increase the government’s environmental performance benefits. For the government and the public, the government subsidies for enterprise carbon label products can promote the public’s purchasing behavior of carbon label products. At the same time, the government’s active responsibility can harvest the prestige of the public. For enterprises and the public, the purchase behavior of carbon label products by the public can provide available and potential market value for enterprises, and the positive R&D behavior of low-carbon technology by enterprises can provide environmental protection value and product value for the public.

The government and enterprises are the main participants in carbon reduction policies, and enterprises and the public are the main components of the low-carbon product market. On this basis, through the above multi-agent driving mechanism and from the perspective of heterogeneity, this section first constructs the “government-enterprise” evolutionary game model, and then explores the interaction between the government and enterprises under the background of CPAN policy and carbon labeling system with consideration of the influence of public sentiment on the strategic choice of the government and enterprises. Secondly, considering the influence of government policy guidance on the strategic choices of enterprises and the public, the “enterprise-public” evolutionary game model is constructed to explore the interaction between enterprises and the public in the carbon label product market. Finally, on the basis of the first two models, the “government-enterprise-public” tripartite evolutionary game model is constructed to explore the influence and the micro driving path among these three subjects.

### 3.1. The Evolutionary Game Model of Government Policy Guidance and Enterprise Low-Carbon Technology R&D Considering Public Sentiment

**Hypothesis** **1.** *In the natural environment without considering other constraints, the subjects of evolutionary game are government and enterprise. Both subjects are bounded rationally, and both have learning ability and their own behavior strategies*.

**Hypothesis** **2.** *The governmental policy guidance and macro-control measures are divided into policy advocacy, incentive measures, and environmental taxes. The enforcement intensity factors are* φ, β, and δ*, and the costs are* φA, βI, and δT, *respectively*.

**Hypothesis** **3.** *The government can choose the strategy of performance and non-performance; when the government chooses the former one, it will gain public prestige* $R_{1}$. *Enterprises can choose low-carbon technology R&D and non-R&D. When enterprises choose the low-carbon technology R&D strategy, the R&D cost is* C. *After completing low-carbon technology R&D, green product market value* V *and public reputation or potential market income* $R_{2}$ *can be obtained. Whether the enterprise low-carbon technology R&D strategy is chosen or not, it will bring the government carbon emissions revenue* $R_{g}$*and loss*$L_{g}$.

**Hypothesis** **4.** x *denotes the probability of government choosing performance strategy;* y *denotes the probability of enterprises choosing low-carbon technology R&D strategy,* x,y∈*[0,1]; and both are functions of time* t. *In order to maximize their own interests, both sides of the game constantly adjust their strategies until they reach their own evolutionary stable strategies;* $x^{*}\;$*and*$y^{*}$*are used to represent the probability choices of the government and enterprises under evolutionary stable strategy (ESS) conditions.*

On the basis of the assumptions listed above, taking into account public sentiment, the revenue matrix of government and enterprise is constructed as shown in Table 1.

When the government chooses the strategy of performing and non-performing responsibilities, the revenues are $\pi_{11}$ and $\pi_{12}$, respectively, and the average expected revenues is ${\;\bar{\pi }}_{1}$. $\pi_{11}$, $\pi_{12}$, ${\;\bar{\pi }}_{1}$ are as follows:

(1) $$\pi_{11}=y(R_{1}\;{+\;R}_{g}\;-\;\varphi A\;-\;\beta I)+\left(1\;-\;y\right)\left({\delta T+R}_{1\;}{-\;\varphi A\;-\;L}_{g}\right)\;$$

(2) $$\pi_{12}{=yR}_{g}{+(1\;-\;y)(-L}_{g})\;$$

(3) $${\;\bar{\pi }}_{1\;}{=x\pi }_{11}{+(1\;-\;x)\pi }_{12}\;$$

As a result, the government’s replication dynamic equation is

(4) $$\frac{dx}{dt}=x\left(\pi_{11}\;{-\;\bar{\pi }}_{1}\right)=x\left(1\;-\;x\right)[-y\left(\beta I+\delta T\right){+R}_{1}+\delta T\;-\;\varphi A]\;$$

Similarly, the revenue $\pi_{21}$, $\pi_{22}$, and the average expectation ${\bar{\pi }}_{2}$ for the enterprise to choose R&D and non-R&D strategies are as follows:

(5) $$\pi_{21\;}=x\left({V+R}_{2}+\beta I\;-\;C\right)+\left(1\;-\;x\right)\left({V+R}_{2}\;-\;C\right)\;$$

(6) $$\pi_{22}=x\left(-\;\delta T\right)\;$$

(7) $${\;\bar{\pi }}_{2\;}{=y\pi }_{21}{+(1\;-\;y)\pi }_{22}\;$$

Thus, the dynamic equation of corporate replication considering public sentiment is

(8) $$\frac{dy}{dt}=y\left(\pi_{21\;}{-\;\bar{\pi }}_{2}\right)=y\left(1\;-\;y\right)[x\left(\beta I+\delta T\right){+V+R}_{2}\;-\;C]\;$$

According to the replication dynamic equation of government, if $y=\frac{R_{1}+\delta T\;-\;\varphi A}{\delta T+\beta I}$, then $\frac{\mathrm{dx}}{\mathrm{dt}}\;\equiv \;0$, and all x levels are ESS; if $y\;>\;\frac{R_{1}+\delta T\;-\;\varphi A}{\delta T+\beta I}$, then $x^{*}=0$ is ESS; if $y\;<\;\frac{R_{1}+\delta T\;-\;\varphi A}{\delta T+\beta I}$, then $x^{*}=1$ is ESS. According to the replicator dynamic equation of the enterprise, if $x=\frac{{C\;-\;(V+R}_{2})}{\delta T+\beta I}$, then $\frac{\mathrm{dy}}{\mathrm{dt}}\;\equiv \;0$, and all y levels are ESS; if $x\;>\;\frac{{C\;-\;(V+R}_{2})}{\delta T+\beta I}$, then $y^{*}=1$ is ESS; if $x\;<\;\frac{{C\;-\;(V+R}_{2})}{\delta T+\beta I}$, then $y^{*}=0$ is ESS.

From the above analysis, it can be seen that ESS of the evolutionary game model is related to the relative size of $R_{1}+\delta T$ and φA and the relative size of C and ${V+R}_{2}$. In the following, we will discuss the influence of the relative size of C and ${P+R}_{2}$ on the ESS in the case of $R_{1}+\delta T\;<\;\varphi A$ and $R_{1\;}+\delta T\;>\;\varphi A$.

**Figure 2:**
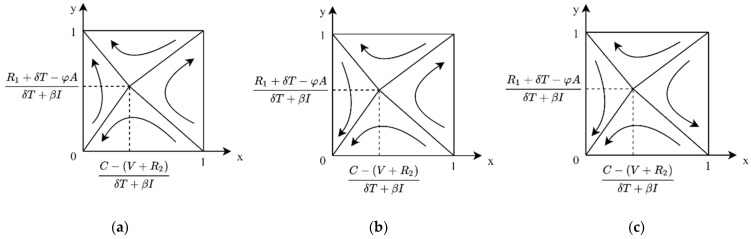
Evolutionary phase diagram of government cost–benefit under $R_{1}+\delta T\;<\;\varphi A$. (**a**) When R&D costs are relatively low. (**b**) When R&D costs are somewhat high. (**c**) When R&D costs are high.

For the government, when $R_{1}+\delta T\;<\;\varphi A$, $\frac{R_{1}+\delta T\;-\;\varphi A}{\delta T+\beta I}\;<\;0$, for any y, there is $-\;y\left(\beta I+\delta T\right){+R}_{1}+\delta T\;-\;\varphi A\;>\;0$, and the non-performance strategy is the government ESS. For the enterprise, there are the following cases:

**Case 1:** If ${C\;<\;V+R}_{2}$, then $\frac{{C\;-\;(V+R}_{2})}{\delta T+\beta I}\;<\;0$, then for any x, $x\left(\beta I+\delta T\right){+V+R}_{2\;}-\;C\;>\;0$, $y^{*}=1$ is the ESS of the enterprise. At this time, the evolutionary phase diagram of both sides of the game is shown in Figure 2a, namely, when there is market value and a positive image of low-carbon products for enterprises, potential market returns greater than R&D costs, government non-performance, and enterprise R&D for the ESS system.

**Case 2:** If ${C\;>\;V+R}_{2}$, there are two cases, $0\;<\;\frac{{C\;-\;(V+R}_{2})}{\delta T+\beta I}\;<\;1$ and $\frac{{C\;-\;(V+R}_{2})}{\delta T+\beta I}\;>\;1$. When C is slightly larger than ${V+R}_{2}$, $0\;<\;\frac{{C\;-\;(V+R}_{2})}{\delta T+\beta I}\;<\;1$, then if $x\;>\;\frac{{C\;-\;(V+R}_{2})}{\delta T+\beta I}$, then $y^{*}=1$ is ESS; if $x\;<\;\frac{{C\;-\;(V+R}_{2})}{\delta T+\beta I}$, then $y^{*}=0$ is ESS. In this case, the evolutionary phase diagram of both sides of the game is shown in Figure 2b. The government non-performance and the enterprise do not have R&D for evolutionary game system ESS. When $C\;\gg {\;V+R}_{2}$, $\frac{{C\;-\;(V+R}_{2})}{\delta T+\beta I}\;>\;1$; for any x, there are $x\left(\beta I+\delta T\right){+V+R}_{2}\;-\;C\;<\;0$. $y^{*}=0$ is the ESS of the enterprise. In this case, the evolutionary phase diagram of both sides of the game is shown in Figure 2c. The government non-performance and the enterprise do not have R&D for evolutionary game system ESS.

Through the above analysis, it can be seen that when the benefits of environmental taxes and public positive emotions towards the government are less than the expenditure of government policy propaganda, the government tends to choose the strategy of non-compliance, and the result of the government–enterprise game depends on the relative size of C and ${V+R}_{2}$. As profit-making organizations, enterprises pay more attention to the direct and potential benefits brought about by products. When there is an impact of low-carbon products on the market value, positive image and potential market return of enterprises is greater than that of R&D costs; even if the government does not perform its duties, enterprises are more inclined to choose R&D strategies, and vice versa.

**Figure 3:**
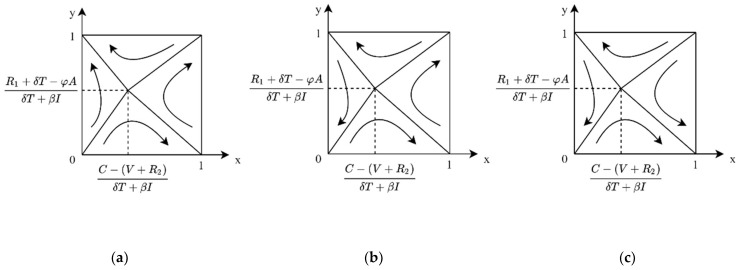
Evolutionary phase diagram of government cost–benefit under $0\;<\;\frac{R_{1}+\delta T\;-\;\varphi A}{\delta T+\beta I}\;<\;1$. (**a**) When R&D costs are relatively low. (**b**) When R&D costs are somewhat high. (**c**) When R&D costs are high.

For the government, when $R_{1}+\delta T\;>\;\varphi A$, there are two cases of $0\;<\;\frac{R_{1}+\delta T\;-\;\varphi A}{\delta T+\beta I}\;<\;1$ and $\frac{R_{1}+\delta T\;-\;\varphi A}{\delta T+\beta I}\;>\;1$. In case $0\;<\;\frac{R_{1}+\delta T\;-\;\varphi A}{\delta T+\beta I}\;<\;1$, if $y\;>\;\frac{R_{1}+\delta T\;-\;\varphi A}{\delta T+\beta I}$, the non-performance strategy is ESS; if $y\;<\;\frac{R_{1}+\delta T\;-\;\varphi A}{\delta T+\beta I}$, the performance strategy is ESS. In the case of $\frac{R_{1}+\delta T\;-\;\varphi A}{\delta T+\beta I}\;>\;1$, $-y\left(\beta I+\delta T\right){+R}_{1}+\delta T\;-\;\varphi A\;<\;0$, the performance strategy is ESS of the government. For the enterprise, there are the following cases:

**Case 1:** When $0\;<\;\frac{R_{1}+\delta T\;-\;\varphi A}{\delta T+\beta I}\;<\;1$, for the enterprise, if ${C\;<\;V+R}_{2}$, then $\frac{{C\;-\;(V+R}_{2})}{\delta T+\beta I}\;<\;0$, then $x\left(\beta I+\delta T\right){+V+R}_{2}\;-\;C\;>\;0$ for any x; $y^{*}=1$ is the ESS of the enterprise. At this time, the evolutionary phase diagram of both sides of the game is shown in Figure 3a. That is, in the case where the benefits of government performance are slightly greater than the costs, when the market value and positive image impact of low-carbon products for the enterprise is greater than the cost of R&D, the government non-performance and the enterprise R&D for the system’s ESS.

**Case 2:** When $0\;<\;\frac{R_{1}+\delta T\;-\;\varphi A}{\delta T+\beta I}\;<\;1$, for the enterprise, if ${C\;>\;V+R}_{2}$, there are two cases of $0\;<\;\frac{{C\;-\;(V+R}_{2})}{\delta T+\beta I}\;<\;1$ and $\frac{{C\;-\;(V+R}_{2})}{\delta T+\beta I}\;>\;1$. When *C* is slightly larger than ${V+R}_{2}$, $0\;<\;\frac{{C\;-\;(V+R}_{2})}{\delta T+\beta I}<\;1$. At this time, if $x\;>\;\frac{{C\;-\;(V+R}_{2})}{\delta T+\beta I}$, then $y^{*}=1$ is ESS, and if $x\;<\;\frac{{C\;-\;(V+R}_{2})}{\delta T+\beta I}$, then $y^{*}=0$ is ESS. In this case, the evolutionary phase diagram of both sides of the game is shown in Figure 3b. The system does not have ESS. When $C\;\gg {\;V+R}_{2}$, $\frac{{C\;-\;(V+R}_{2})}{\delta T+\beta I}\;>\;1$, at this time, for any x, we have $x\left(\beta I+\delta T\right){+V+R}_{2}\;-\;C\;<\;0$, and $y^{*}=0$ is the ESS of the enterprise. In this case, the evolutionary phase diagram of both sides of the game is shown in Figure 3c. The government non-performance and the enterprise R&D for the system’s ESS. 

**Figure 4:**
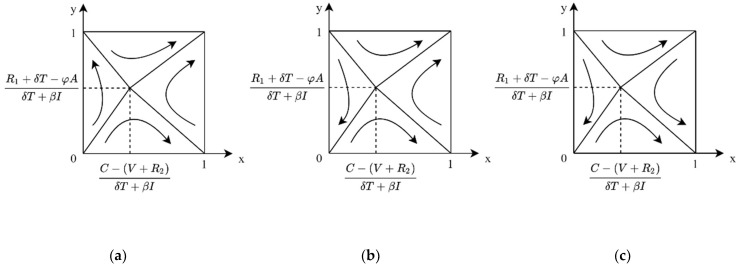
Evolutionary phase diagram of government cost–benefit under $\frac{R_{1}+\delta T\;-\;\varphi A}{\delta T+\beta I}\;>\;1$. (**a**) When R&D costs are relatively low. (**b**) When R&D costs are somewhat high. (**c**) When R&D costs are high.

**Case 3:** When $\frac{R_{1}+\delta T\;-\;\varphi A}{\delta T+\beta I}\;>\;1$, for the enterprise, if ${C\;<\;V+R}_{2}$, then $\frac{{C\;-\;(V+R}_{2})}{\delta T+\beta I}\;<\;0$. At this time, for any x, we have $x\left(\beta I+\delta T\right){+V+R}_{2}\;-\;C\;>\;0$, and $y^{*}=1$ is the ESS of the enterprise. At this time, the evolutionary phase diagram of both sides of the game is shown in Figure 4a, i.e., when the market value and positive image impact of low-carbon products for the enterprise is greater than the cost of R&D, the government performance and the enterprise R&D is the ESS of the system. 

**Case 4:** When $\frac{R_{1}+\delta T\;-\;\varphi A}{\delta T+\beta I}\;>\;1$, for the enterprise, if ${C\;>\;V+R}_{2}$, there are two cases of $0\;<\;\frac{{C\;-\;(V+R}_{2})}{\delta T+\beta I}\;<\;1$ and $\frac{{C\;-\;(V+R}_{2})}{\delta T+\beta I}\;>\;1$. When C is slightly larger than ${V+R}_{2}$, $0\;<\;\frac{{C\;-\;(V+R}_{2})}{\delta T+\beta I}\;<\;1$. At this time, if $x\;>\;\frac{{C\;-\;(V+R}_{2})}{\delta T+\beta I}$, then $y^{*}=1$ is ESS, and if $x\;<\;\frac{{C\;-\;(V+R}_{2})}{\delta T+\beta I}$, then $y^{*}=0$ is ESS. In this case, the evolutionary phase diagram of the game is shown in Figure 4b. The government performance and the enterprise R&D is the ESS of the evolutionary game system. When $C\;\gg {\;V+R}_{2}$, $\frac{{C\;-\;(V+R}_{2})}{\delta T+\beta I}\;>\;1$. At this time, for any x, we have $x\left(\beta I+\delta T\right){+V+R}_{2}\;-\;C\;<\;0$, and $y^{*}=0$ is the ESS of the enterprise. In this case, the evolutionary phase diagram of the game is shown in Figure 4c. The government performance and the enterprise does not have R&D as the ESS of the evolutionary game system.

The above analysis shows that when environmental taxes, fees, and positive public sentiment bring more benefits to the government than the expenses of government policy propaganda, the results of the “government–enterprise” game mainly depend on the relative sizes of C and ${V+R}_{2}$. Moreover, when the market value and positive image of low-carbon products for the enterprise and the potential market value influence are greater than R&D costs, the enterprise is more inclined to choose the R&D strategy. However, there are still cases that the government chooses the performance strategy and the enterprise chooses the R&D strategy when the influence of market value, positive image, and potential market value is less than R&D cost, which indicates that effective government policy guidance is a key factor in promoting the enterprise’s participation in low-carbon technology R&D.

In summary, in the evolutionary game model of “government–enterprise”, whether the government performance and whether the enterprise engages in low-carbon technology R&D is influenced by public sentiment in addition to financial revenue, expenditure, and cost–benefit. When the high cost of policy promotion and R&D does not allow the government to choose the performance strategy and the enterprise to choose the R&D strategy, the strong public sentiment will monitor the government and make the government choose the performance strategy, while the enterprise will also see the potential benefits and choose the R&D strategy. In addition, the effective guidance of government policy provides the possibility for the enterprise to choose R&D strategy with less cost–benefit, which indicates that government guidance is a key factor for enterprises to change their short-sighted behavior and engage in low-carbon technology R&D.

### 3.2. An Evolutionary Game Model of Public Purchasing Behavior of Carbon-Labeled Products and Enterprise Low-Carbon Technology R&D Considering Government Guidance

**Hypothesis** **5.** *The government’s guiding behavior is divided into guiding the public to purchase carbon labeled products and guiding the enterprise to conduct low-carbon technology R&D. The government subsidizes the public who purchase the enterprise’s carbon-labeled products with an implementation intensity factor of* θ*and a subsidy cost of* θS*. In a natural environment without considering other constraints, the two subjects of the evolutionary game are the public and the enterprise. Both subjects are finite rational actors, and both have learning ability and respective behavioral strategies.*

**Hypothesis** **6.** *The public can choose between purchasing and not purchasing the carbon-labeled products; the market price of the carbon-labeled product is* P, *and the product provides the public with a product value of* $V_{c}$.

**Hypothesis** **7.** *The enterprise can choose between low-carbon technology R&D and non-R&D. Accordingly, the choice of enterprise’s strategies will result in low-carbon environmental gains* $R_{c}$*or losses* $L_{c}$*for the public. In addition, if the public is willing to purchase carbon-labeled products and the enterprise chooses the non-R&D strategy, the enterprise will lose opportunity cost*U.

**Hypothesis** **8.** y*denotes the probability that the enterprise chooses a low-carbon technology R&D strategy, and* z *denotes the probability that the public chooses the purchasing carbon-labeled product strategy*. y,z∈*[0,1], and both are functions of time* t. *In order to maximize their own interests, both sides of the game continuously adjust their strategies until they reach their respective evolutionary stability strategies.* $y^{*}$*and*$z^{*}$ *denote the probability choices of the enterprise and the public under ESS conditions, respectively, and the rest of the symbols are the same as above*.

On the basis of the above assumptions, taking into account government guidance, the revenue matrix of enterprise and the public is constructed as shown in Table 2.

When the public chooses to purchase and does not purchase, the returns are $\pi_{31}$ and $\pi_{32}$, respectively, and the average expected return is ${\;\bar{\pi }}_{3}$. $\pi_{31}$, $\pi_{32}$, ${\;\bar{\pi }}_{3}$ are as follows:

(9) $$\pi_{31}=y\left(V_{c}{+R}_{c}+\theta S\;-\;P\right)+\left(1\;-\;y\right)\left(V_{c}\;{-\;P\;-\;L}_{c}\right)\;$$

(10) $$\pi_{32}{=yR}_{c}{+(1\;-\;y)(-L}_{c})\;$$

(11) $${\;\bar{\pi }}_{3}{=z\pi }_{31}+\left(1\;-\;z\right)\pi_{32}\;$$

Thus, the replicator dynamic equation of the public under the guidance of the government is as follows:

(12) $$\frac{dz}{dt}=z\left(\pi_{31}{\;-\;\bar{\pi }}_{3}\right)=z\left(1\;-\;z\right){[y\theta S+V}_{c}\;-\;P]\;$$

Similarly, the income $\pi_{41}$ and $\pi_{42}$ of enterprise when it chooses R&D and non-R&D strategies and their average expectation ${\;\bar{\pi }}_{4}$ are as follows:

(13) $$\pi_{41}=z\left({V+R}_{2}+\beta I\;-\;C\right)+\left(1\;-\;z\right)\left({\beta I+R}_{2}\;-\;C\right)\;$$

(14) $$\pi_{42}=z\left(-\delta T\;-\;U\right)+(1\;-\;z)(-\delta T)\;$$

(15) $${\;\bar{\pi }}_{4}{=y\pi }_{41}{+(1\;-\;y)\pi }_{42}\;$$

Thus, under the guidance of government, the dynamic equation of enterprise replication is

(16) $$\frac{dy}{dt}=y\left(\pi_{41}{\;-\;\bar{\pi }}_{4}\right)=y\left(1\;-\;y\right)[z\left(V+U\right){+\beta I+\delta T+R}_{2}\;-\;C]\;$$

According to the public replication dynamic equation, if $y=\frac{{P\;-\;V}_{c}}{\theta S}$, then $\frac{\mathrm{dz}}{\mathrm{dt}}\;\equiv \;0$, where all z levels are ESS; if $y\;>\;\frac{{P\;-\;V}_{c}}{\theta S}$, then $z^{*}=1$ is ESS; if $y\;<\;\frac{{P\;-\;V}_{c}}{\theta S}$, then $z^{*}=0$ is ESS. According to the replicator dynamic equation of enterprise, if $z=\frac{{C\;-\;(R}_{2}+\delta T+\beta I)}{V+U}$, then $\frac{\mathrm{dy}}{\mathrm{dt}}\;\equiv \;0$, and all y levels are ESS; if $z\;>\;\frac{{C\;-\;(R}_{2}+\delta T+\beta I)}{V+U}$, then $y^{*}=1$ is ESS; if $z\;<\;\frac{{C\;-\;(R}_{2}+\delta T+\beta I)}{V+U}$, then $y^{*}=0$ is ESS. 

From the above analysis, it can be seen that ESS of the evolutionary game model is related to the relative size of $V_{c}$ and P and the relative size of C and ${\beta I+\delta T+R}_{2}$, where βI+δT represents the government’s supervision on enterprise. In the following section, we discuss the influence of the relative size of $V_{c}$ and *P* on the ESS for ${C\;<\;\beta I+\delta T+R}_{2}$ and ${C\;>\;\beta I+\delta T+R}_{2}$. 

**Figure 5:**
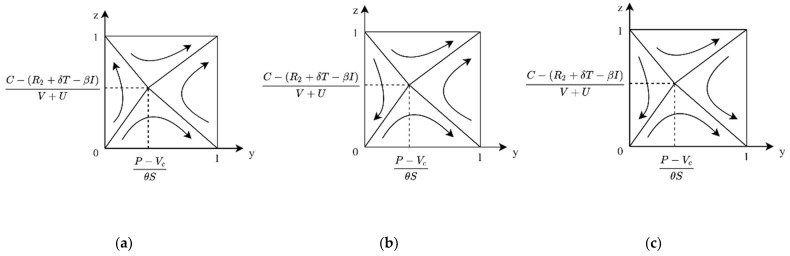
Evolutionary phase diagram of cost–benefit of enterprise under ${C\;<\;\beta I+\delta T+R}_{2}$. (**a**) When the price is lower than the product value. (**b**) When the price is slightly higher than the product value. (**c**) When prices are much higher than the product value.

For the enterprise, when ${C\;<\;\beta I+\delta T+R}_{2}$, $\frac{{C\;-\;(R}_{2\;}+\delta T+\beta I)}{V+U}\;<\;0$; for any z, there is $z\left(V+U\right){+\beta I+\delta T+R}_{2}\;-\;C\;>\;0$, the R&D strategy is ESS. For the public, there are the following cases:

**Case 1:** If ${P\;<\;V}_{c}$, then $\frac{{P\;-\;V}_{c}}{\theta S}\;<\;0$, then for any y, there is ${y\theta S+V}_{c}\;-\;P\;>\;0$, and $z^{*}=1$ for the public ESS. At this time, the evolutionary phase diagram of both sides of the game is shown in Figure 5a. In the case of low enterprise R&D cost, when the product value of carbon label products for consumers is greater than the market price, enterprise R&D and public purchase is the ESS for the system.

**Case 2:** If ${P\;>\;V}_{c}$, there are two cases $0\;<\;\frac{{P\;-\;V}_{c}}{\theta S}\;<\;1$ and $\frac{{P\;-\;V}_{c}}{\theta S}\;>\;1$. When P is slightly larger than $V_{c}$ or the government subsidy θS is large enough, $0\;<\;\frac{{P\;-\;V}_{c}}{\theta S}\;<\;1$; at this time, if $y\;>\;\frac{{P\;-\;V}_{c}}{\theta S}$, $z^{*}=1$ is ESS, and if $y\;<\;\frac{{P\;-\;V}_{c}}{\theta S}$, $z^{*}=0$ is ESS. In this case, the evolutionary phase diagram of both sides of the game is shown in Figure 5b, where enterprise R&D and public purchase are ESS of the evolutionary game system. When $P\;\gg {\;V}_{c}$, $\frac{{P\;-\;V}_{c}}{\theta S}\;>\;1$, at this point, for any y, there are ${y\theta S+V}_{c}\;-\;P\;<\;0$, $z^{*}=0$ for the ESS of public. In this case, the evolutionary phase diagram of both sides of the game is shown in Figure 5c, where enterprise R&D and public non-purchase are the ESS of evolutionary game system.

Through the above analysis, it can be seen that when the government’s policy guidance and supervision are strong and the public’s support for carbon label products is high, the enterprise is more inclined to choose the R&D strategy, and the results of the game of “enterprise–public” mainly depend on the relative size of P and $V_{c}$. Although government guidance will have a certain impact on the public’s carbon label purchase behavior, the public, as a consumer group of low-carbon technology products, pays more attention to the product value of the products provided by the enterprise, so when the product value of carbon label products is greater than the market price, the public is more inclined to choose the purchase strategy, while in the opposite case, the public is more inclined to choose the non-purchase strategy.

For the enterprise, when ${C\;>\;\beta I+\delta T+R}_{2}$, there are $0\;<\;\frac{{C\;-\;(R}_{2}+\delta T+\beta I)}{V+U}\;<\;1$ and $\frac{{C\;-\;(R}_{2}+\delta T+\beta I)}{V+U}\;>\;1$, two cases.

**Figure 6:**
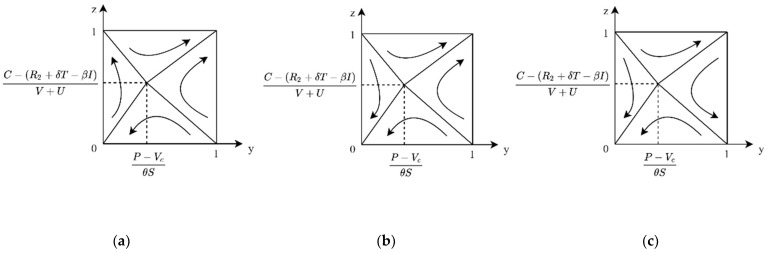
Evolutionary phase diagram of cost–benefit of enterprise under $0\;<\;\frac{{C\;-\;(R}_{2}+\delta T+\beta I)}{V+U}\;<\;1$. (**a**) When the price is lower than the product value. (**b**) When the price is slightly higher than the product value. (**c**) When prices are much higher than the product value.

In case $0\;<\;\frac{{C\;-\;(R}_{2\;}+\delta T+\beta I)}{V+U}\;<\;1$, if $z\;>\;\frac{{C\;-\;(R}_{2}+\delta T+\beta I)}{V+U}$, then the R&D strategy is ESS; if $z\;<\;\frac{{C\;-\;(R}_{2}+\delta T+\beta I)}{V+U}$, then ESS is non-R&D strategy. In the case of $\frac{{C\;-\;(R}_{2}+\delta T+\beta I)}{V+U}\;>\;1$, there are $z\left(V+U\right){+\beta I+\delta T+R}_{2}\;-\;C\;<\;0$, where the non-R&D strategy is ESS for the enterprise. For the public, there are the following cases:

**Case 1:** When $0\;<\;\frac{C\;-\;\left(R_{2}+\delta T+\beta I\right)}{V+U}\;<\;1$, for the public, if ${P\;<\;V}_{c}$, then $\frac{{P\;-\;V}_{c}}{\theta S}\;<\;0$, for any y, ${y\theta S+V}_{c}\;-\;P\;>\;0$, and $z^{*}=1$ is public’s ESS. At this time, the evolutionary phase diagram of both sides of the game is shown in Figure 6a. In the case of slightly higher R&D costs, when the value of carbon label products for the public is greater than the market price, the enterprise R&D and public purchase is the ESS of the system.

**Case 2:** When $0\;<\;\frac{{C\;-\;(R}_{2}+\delta T+\beta I)}{V+U}\;<\;1$, for the public, if ${P\;>\;V}_{c}$, there are two cases $0\;<\;\frac{{P\;-\;V}_{c}}{\theta S}\;<\;1$ and $\frac{{P\;-\;V}_{c}}{\theta S}\;>\;1$. When P is slightly larger than $V_{c}$, $0\;<\;\frac{{P\;-\;V}_{c}}{\theta S}\;<\;1$, then, if $y\;>\frac{{\;P\;-\;V}_{c}}{\theta S}$, $z^{*}=1$ is ESS, if $y\;<\;\frac{{P\;-\;V}_{c}}{\theta S}$, and $z^{*}=0$ is ESS. In this case, the evolutionary phase diagram of both sides of the game is shown in Figure 6b. The system has two ESSs: enterprise R&D, public purchase, and enterprise not R&D, public not purchase. When $P\;\gg {\;V}_{c}$, $\frac{{P\;-\;V}_{c}}{\theta S}\;>\;1$, at this point, for any y, there are ${y\theta S+V}_{c}\;-\;P\;<\;0$, $z^{*}=0$ for the public’s ESS. In this case, the evolutionary phase diagram of both sides of the game is shown in Figure 6c, where enterprise does not have R&D, and the public not purchasing is the ESS of the system.

**Figure 7:**
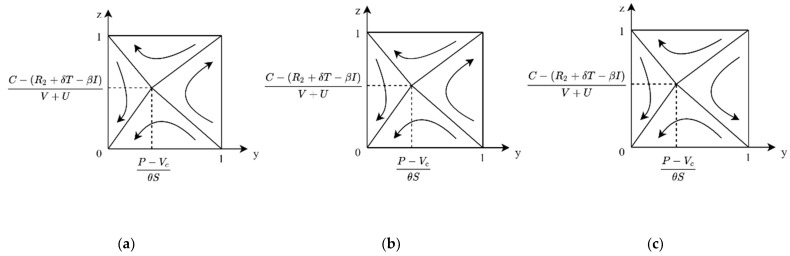
Evolutionary phase diagram of cost–benefit of enterprise under $\frac{{C\;-\;(R}_{2}+\delta T+\beta I)}{V+U}\;>\;1$. (**a**) When the price is lower than the product value. (**b**) When the price is slightly higher than the product value. (**c**) When prices are much higher than the product value.

**Case 3:** When $\frac{{C\;-\;(R}_{2}+\delta T+\beta I)}{V+U}\;>\;1$, for the public, if ${P\;<\;V}_{c}$, then $\frac{{P\;-\;V}_{c}}{\theta S}\;<\;0$; at this time, for any y, there is ${y\theta S+V}_{c}\;-\;P\;>\;0$, and $z^{*\;}=1$ for the public’s ESS. At this time, the evolutionary phase diagram of both sides of the game is shown in Figure 7a. In the case of high R&D costs, when the value of carbon label products for the public is greater than the market price, the enterprise does not have R&D, and public purchase is the ESS of the system.

**Case 4:** When $\frac{{C\;-\;(R}_{2}+\delta T+\beta I)}{V+U}\;>\;1$, for the public, if ${P\;>\;V}_{c}$, there are two cases $0\;<\;\frac{{P\;-\;V}_{c}}{\theta S}\;<\;1$ and $\frac{{P\;-\;V}_{c}}{\theta S}\;>\;1$. When P is slightly larger than $V_{c}$, $0\;<\;\frac{{P\;-\;V}_{c}}{\theta S}\;<\;1$, then if $y\;>\;\frac{{P\;-\;V}_{c}}{\theta S}$, $z^{*}=1$ is ESS; if $y\;<\;\frac{{P\;-\;V}_{c}}{\theta S}$, then $z^{*}=0$ is ESS. In this case, the evolutionary phase diagram of the game is as shown in Figure 7b. The enterprise does not have R&D, and the public not purchasing is the ESS for the system. When $P\;\gg {\;V}_{c}$, $\frac{{P\;-\;V}_{c}}{\theta S}\;>\;1$, at this point, for any y, there are ${y\theta S+V}_{c}\;-\;P\;<\;0$, and $z^{*}=0$ for the public ESS. In this case, the evolutionary phase diagram of the game is as shown in Figure 7c. The enterprise does not have R&D, and the public not purchasing is the ESS of the system.

Through the above analysis, it can be seen that when the R&D cost of low-carbon technology of enterprise is higher than that of government supervision and the positive impact of carbon label products on the enterprise, the enterprise is more inclined to choose the non-R&D strategy. However, the market value of carbon label products and the opportunity cost of abandoning R&D provide the possibility for enterprise to choose the R&D strategy. At this time, the results of the “enterprise–public” game mainly depend on the relative size of P and $V_{c}$. As consumers of carbon label products, the public’s choice of whether to purchase carbon label products is not only affected by the government’s guiding behavior but also depends on the price and value of the products. When the value of carbon label products for the public is greater than the price, the public is more inclined to buy.

In summary, in the “enterprise–public” evolutionary game model, the government’s effective guidance has a positive effect on the enterprise’s choice of low-carbon technology R&D strategy and the public’s choice of purchasing carbon label products strategy, and for the game subjects of enterprises and the public, there is a mutual influence relationship between their strategy choices: if the market value and potential value of carbon label products for the enterprise is high, and the opportunity cost of the enterprise to abandon low-carbon technology R&D is high, the enterprise is more inclined to choose the R&D strategy. If the value of carbon label products is high or the purchase cost is low, the public is more inclined to choose the purchase strategy.

### 3.3. Evolutionary Game Model of Government Guidance, Enterprise Low-Carbon Technology R&D, and Public Carbon Label Purchase Behavior

In the first two parts, this paper discussed the interaction between the government and enterprises, both of which are the main participants in carbon reduction policies, and also discussed the interaction between enterprises and the public, which are the main components of the carbon label product market. However, under the CPAN policy, the interests of the three parties in the carbon label system are not strictly the same. For the government, since the R&D of low-carbon technologies and the CPAN policy are crucial to the country, it has sufficient motivation to guide enterprises to carry out low-carbon technology R&D and create a good market environment for carbon label products. For enterprises, they are more likely to consider low-carbon technology R&D from the perspective of cost–benefit. For the public, as consumers of carbon label products, they are more likely from the perspective of product value and price to consider whether or not to buy carbon label products. In the above section, we concluded that government guidance is the key factor for enterprise to carry out low-carbon technology R&D, and the enterprise’s low-carbon technology R&D behavior interacts with the public’s purchase of carbon label products. On this basis, this section constructs the “government–enterprise–public” tripartite evolutionary game model to explore the interaction between these three subjects more intuitively. 

#### 3.3.1. Model Establishment

**Hypothesis** **9.** *In the natural environment without considering other constraints, the tripartite subjects of the evolutionary game are the government, the enterprise, and the public. The tripartite subjects are all bounded rationally, having learning ability and their own behavioral strategies*.

**Hypothesis** **10.** *The government can choose the strategy of performance or non-performance*; x*represents the probability of the government choosing the strategy of performance. The enterprise can choose R&D and non-R&D strategies;*y*represents the probability of the enterprise choosing the low-carbon technology R&D strategy. Purchasing and non-purchasing are the public’s choice strategies*. z*is the probability of the public choosing to purchase low-carbon products*. x, y, z ∈ *[0,1], and all are time*t*functions. In order to maximize their own interests, the three parties in this game constantly adjust their strategies until they reach their own evolutionary stable strategies.*$x^{*}$, $y^{*}$*, and*$z^{*}$*are used to represent the probability of choices of the government, the enterprise, and the public under ESS conditions, respectively. Their symbols are the same as above*.

On the basis of the above assumptions, a tripartite evolutionary game revenue matrix of government, enterprise, and the public was constructed, as shown in Table 3.

The government’s revenues are $\pi_{11}$, and $\pi_{12}$ when choosing the strategy of performance and non-performance, respectively, and the average expected revenue is ${\;\bar{\pi }}_{1}$. $\pi_{11}$, $\pi_{12}$, ${\;\bar{\pi }}_{1}$ are as follows:

(17) $$\pi_{11}=z\left[y\left(R_{1}{+R}_{g}\;-\;\varphi A\;-\;\beta I\;-\;\theta S\right)+\left(1\;-\;y\right)\left({\delta T+R}_{1}{\;-\;\varphi A\;-\;L}_{g}\right)\right]+\left(1\;-\;z\right)[y\left(R_{1}{+R}_{g}\;-\;\varphi A\;-\;\beta I\right){+(1\;-\;y)(\delta T+R}_{1}{\;-\;\varphi A\;-\;L}_{g})]\;$$

(18) $$\pi_{12}=z\left[{\mathrm{yR}}_{g}+\left(1\;-\;y\right)\left({-L}_{g}\right)\right]+\left(1\;-\;z\right){[\mathrm{yR}}_{g}+\left(1\;-\;y\right)\left({-L}_{g}\right)]\;$$

(19) $${\;\bar{\pi }}_{1}{=x\pi }_{11}{+(1\;-\;x)\pi }_{12}\;$$

Thus, the government’s replication dynamic equation is

(20) $$\frac{dx}{dt}=x\left(\pi_{11}{\;-\;\bar{\pi }}_{1}\right)=x\left(1\;-\;x\right)[-y\left(z\theta S+\beta I+\delta T\right){+\delta T+R}_{1}\;-\;\varphi A]\;$$

When the enterprise chooses R&D and non-R&D strategies, the revenue $\pi_{21}$, $\pi_{22}$, and the average expectation ${\;\bar{\pi }}_{2}$ are as follows:

(21) $$\pi_{21}=z\left[x\left({V+R}_{2}+\beta I\;-\;C\right)+\left(1\;-\;x\right)\left({V+R}_{2}\;-\;C\right)\right]+\left(1\;-\;z\right)[x\left({V+R}_{2}\;-\;C\right)+(1\;-\;x)\left(R_{2}\;-\;C\right)]\;$$

(22) $$\pi_{22}=z[x\left(-\delta T\;-\;U\right)+\left(1\;-\;x\right)\left(-U\right)]+x\left(1\;-\;z\right)\left(-\delta T\right)\;$$

(23) $${\;\bar{\pi }}_{2}{=y\pi }_{21}{+(1\;-\;y)\pi }_{22}\;$$

Thus, the dynamic equation of enterprise replication is

(24) $$\frac{dy}{dt}=y\left(\pi_{21}\;-{\bar{\;\pi }}_{2}\right)=y\left(1\;-\;y\right)[z\left(V+U\right)+x\left(V+\delta T\right)+\mathrm{zx}\left(\beta I\;-\;V\right){+R}_{2}\;-\;C]\;$$

The revenue $\pi_{31}$, $\pi_{32}$ and the average expectation ${\;\bar{\pi }}_{3}$ when the public chooses to purchase and does not purchase are as follows:

(25) $$\pi_{31}=x\left[y\left(V_{c}{+R}_{c}+\theta S\;-\;P\right)+\left(1\;-\;y\right)\left(V_{c}{\;-\;P\;-\;L}_{c}\right)\right]+\left(1\;-\;x\right)[y\left(V_{c}{+R}_{c}\;-\;P\right)+\left(1\;-\;y\right)\left(V_{c}{\;-\;P\;-\;L}_{c}\right)]\;$$

(26) $$\pi_{32}=x\left[{\mathrm{yR}}_{c}+\left(1\;-\;y\right)\left({-L}_{c}\right)\right]+\left(1\;-\;x\right){[\mathrm{yR}}_{c}{+(1\;-\;y)(-L}_{c})]\;$$

(27) $${\;\bar{\pi }}_{2}{=y\pi }_{21}{+(1\;-\;y)\pi }_{22}\;$$

Thus, the public replication dynamic equation is

(28) $$\frac{dz}{dt}=z\left(\pi_{31}{\;-\;\bar{\pi }}_{3}\right)=z\left(1\;-\;z\right){[\mathrm{xy}\theta S+V}_{c}\;-\;P]\;$$

Among them, the evolution phase diagram of government stability is related to curve $-y\left(z\theta S+\beta I+\delta T\right){+\delta T+R}_{1}\;-\;\varphi A=0$, the evolution phase diagram of enterprise stability is related to curve $z\left(V+U\right)+x\left(V+\delta T\right)+\mathrm{zx}\left(\beta I\;-\;V\right){+R}_{2}\;-\;C=0$, and the evolution phase diagram of public stability is related to curve ${\mathrm{xy}\theta S+V}_{c}\;-\;P=0$.

#### 3.3.2. Stability Analysis

Let $F(x)=\frac{dx}{dt}=0,\;F(y)=\frac{dy}{dt}=0$*, F(z) =* $\frac{dz}{dt}=0$, and then (0, 0, 0), (1, 0, 0), (0, 1, 0), (0, 0, 1), (1, 1, 0), (1, 0, 1), (0, 1, 1), (1, 1, 1) are the eight fixed local equilibrium points of the system. The boundary of the solution domain Ω of the tripartite evolutionary game is composed of the above eight fixed local equilibrium points, namely, $\Omega =\{\left(x,y,z\right)\;|\;0\;<\;x\;<\;1,\;0\;<\;y\;<\;1,\;0\;<\;z\;<\;1\}$. In the solution domain Ω of the tripartite evolution, there is also an equilibrium point (*x, y, z*), which satisfies

(29) $$\left\{\begin{array}{c} -y\;\left(z\theta S+\beta I+\delta T\right){+\delta T+R}_{1}\;-\;\varphi A=0\\ z\left(V+U\right)+x\left(V+\delta T\right)+\mathrm{zx}\left(\beta I\;-\;V\right){+R}_{2}\;-\;C=0\\ {\mathrm{xy}\theta S+V}_{c}\;-\;P=0 \end{array}\right.$$

The Jacobian matrix of the system can be obtained by calculating the partial derivatives of *F (x)*, *F (y)*, and *F (z)* with respect to *x, y,* and *z*:

$$J=\left[\begin{array}{ccc} \frac{dF\left(x\right)}{dx} & \frac{dF\left(x\right)}{dy} & \frac{dF\left(x\right)}{dz}\\ \frac{dF\left(y\right)}{dx} & \frac{dF\left(y\right)}{dy} & \frac{dF\left(y\right)}{dz}\\ \frac{dF\left(z\right)}{dx} & \frac{dF\left(z\right)}{dy} & \frac{dF\left(z\right)}{dz} \end{array}\right]$$

(30) $$=\left[\begin{array}{ccc} (1-2x)[-y\left(z\theta S+\beta I+\delta T\right){+\delta T+R}_{1}-\varphi A] & -x(1-x)\left(z\theta S+\beta I+\delta T\right) & 0\\ \mathrm{zy}(1-y)\left(V+\delta T\right)\left(\beta I-V\right) & (1-2y)[z\left(V+U\right)+x\left(V+\delta T\right)+\mathrm{zx}\left(\beta I-V\right){+R}_{2}-C] & y(1-y)(V+U)\\ \mathrm{yz}\theta S(1-z) & \mathrm{xz}\theta S(1-z) & {(1-2z)(\mathrm{xy}\theta S+V}_{c}-P) \end{array}\right]$$

Using the Lyapunov indirect method [38], when the eigenvalues of the Jacobian matrix have negative real part, the corresponding equilibrium point is ESS. Otherwise, the corresponding equilibrium point is saddle point. Thus, the following proposition can be obtained:

**Proposition** **1** . *If* ${\delta T+R}_{1}\;<\;\varphi A$, $R_{2}\;<\;C\;$*and* $V_{c}\;<\;P$ *are established at the same time, then point (0, 0, 0) is the ESS of the system. Otherwise, point (0, 0, 0) is a saddle point of the system.*

**Proof** **1**. When the equilibrium point is (0, 0, 0), the Jacobian matrix is

$$\left[\begin{array}{ccc} {\delta T+R}_{1}\;-\;\varphi A & 0 & 0\\ 0 & R_{2}\;-\;C & 0\\ 0 & 0 & V_{c}\;-\;P \end{array}\right]$$

The eigenvalue $\lambda =\left[\begin{array}{c} {\delta T+R}_{1\;}-\;\varphi A\\ R_{2\;}-\;C\\ V_{c}\;-\;P \end{array}\right]$ can be obtained by solving this point. If ${\delta T+R}_{1}\;<\;\varphi A$, $R_{2}\;<\;C$ and $V_{c}\;<\;P$ are established at the same time, and then point (0, 0, 0) is the ESS of the system. If one or more eigenvalues are not negative, point (0, 0, 0) is a saddle point of the system.  □

**Explanation** **1.** *In the equilibrium state of Proposition 1, the stability strategies of the government, enterprise, and the public are non-performance, non-R&D, and non-purchase, respectively. This is a bad equilibrium state, not the problem we want to study*.

**Proposition** **2.** *If*${\varphi A+\beta I\;>\;R}_{1}$, $R_{2}\;>\;C$*and*$V_{c}\;<\;P$*are established at the same time, then point (0, 1, 0) is the ESS of the system. Otherwise, point (0, 1, 0) is a saddle point of the system*.

**Proof** **2**. When the equilibrium point is (0, 1, 0), the Jacobian matrix is

$$\left[\begin{array}{ccc} R_{1}\;-\;\beta I\;-\;\varphi A & 0 & 0\\ 0 & {C\;-\;R}_{2} & 0\\ 0 & 0 & V_{c}\;-\;P \end{array}\right]$$

The eigenvalue $\lambda =\left[\begin{array}{c} R_{1\;}-\;\beta I\;-\;\varphi A\\ {C\;-\;R}_{2}\\ V_{c}\;-\;P \end{array}\right]$ can be obtained by solving this point. If ${\varphi A+\beta I\;>\;R}_{1}$, $R_{2}\;>\;C$ and $V_{c}\;<\;P$ are established at the same time, then point (0, 1, 0) is the ESS of the system. If one or more eigenvalues are not negative, point (0, 1, 0) is a saddle point of the system.  □

**Explanation** **2.** *Under the equilibrium state of Proposition 2, the stable strategies of the government, enterprise, and the public are, respectively, non-performance, R&D and non-purchase. In this case, enterprises are not affected by government policies and public awareness of purchasing carbon label products, and actively choose a low-carbon technology R&D strategy. This is an ideal state that is not in line with the current situation of most Chinese enterprises, which are seeking rapid expansion, even at the expense of polluting the environment, so this situation is not worth discussing*.

**Proposition** **3.** *If*${\delta T+R}_{1}\;<\;\varphi A$, ${V+U+R}_{2}\;<\;C$*and*$V_{c}\;>\;P$*are established at the same time, then point (0, 0, 1) is the ESS of the system. Otherwise, point (0, 0, 1) is a saddle point of the system*.

**Proof** **3.** When the equilibrium point is (0, 0, 1), the Jacobian matrix is

$$\left[\begin{array}{ccc} {\delta T+R}_{1\;}-\;\varphi A & 0 & 0\\ 0 & {V+U+R}_{2}\;-\;C & 0\\ 0 & 0 & {P\;-\;V}_{c} \end{array}\right]$$

The eigenvalue $\lambda =\left[\begin{array}{c} {\delta T+R}_{1}\;-\;\varphi A\\ {V+U+R}_{2}\;-\;C\\ {P\;-\;V}_{c} \end{array}\right]$ can be obtained by solving this point. If ${\delta T+R}_{1}\;<\;\varphi A$, ${V+U+R}_{2}\;<\;C$ and $V_{c}\;>\;P$ are established at the same time, then point (0, 0, 1) is the ESS of the system. If one or more eigenvalues are not negative, point (0, 0, 1) is a saddle point of the system.  □

**Explanation** **3.** *Under the equilibrium state of Proposition 3, the stability strategies of the government, enterprise, and the public are non-performance, non-R&D, and purchase, respectively. In this case, the public has a high awareness of environmental protection, buying carbon label products actively. However, in real life, the government’s non-performance strategy and the enterprise’s non-R&D strategy will hit the public’s enthusiasm to buy carbon label products, ultimately leading to the public’s choice of non-purchase strategy. In this case, this system will become the state of proposition 1, which is not the problem we want to study*.

**Proposition** **4.** If ${\varphi A+\beta I\;<\;R}_{1}$, ${C\;<\;V+\delta T+R}_{2}$ *and* ${P\;<\;\theta S+V}_{c}$ *are established at the same time, then point (1, 1, 0) is the ESS of the system. Otherwise, point (1, 1, 0) is a saddle point of the system*.

**Proof** **4.** When the equilibrium point is (1, 1, 0), the Jacobian matrix is

$$\left[\begin{array}{ccc} {\beta I\;-R}_{1}+\varphi A & 0 & 0\\ 0 & {C\;-\;V+\delta T+R}_{2} & 0\\ 0 & 0 & {P\;-\;(\theta S+V}_{c}) \end{array}\right]$$

The eigenvalue $\lambda =\left[\begin{array}{c} {\beta I\;-\;R}_{1}+\varphi A\\ {C\;-\;V+\delta T+R}_{2}\\ {P\;-\;(\theta S+V}_{c}) \end{array}\right]$ can be obtained by solving this point. If ${\varphi A+\beta I\;<\;R}_{1}$, ${C\;<\;V+\delta T+R}_{2}$ and ${P\;<\;\theta S+V}_{c}$ are established at the same time, then point (1, 1, 0) is the ESS of the system. If one or more eigenvalues are not negative, point (1, 1, 0) is a saddle point of the system.  □

**Explanation** **4.** *Under the equilibrium state of Proposition 4, the stable strategies of the government, enterprise, and the public are, respectively, performance, R&D, and non-purchase. In this case, due to the government’s greater regulatory efforts, enterprises have chosen the low-carbon technology R&D strategy, but the public’s low willingness to buy carbon label products is low, which will to some extent undermine the enthusiasm of enterprises to carry out low-carbon technology R&D, and is not conducive to the promotion of CPAN policy and the carbon label system in China. This is an unsatisfactory state, so it is not discussed here*.

**Proposition** **5.** *If*${\varphi A\;<\;\delta T+R}_{1}$, ${C\;>\;V+\delta T+U+\beta I+R}_{2}$*and*${P\;<\;V}_{c}$*are established at the same time, then point (1, 0, 1) is the ESS of the system. Otherwise, point (1, 0, 1) is a saddle point of the system*.

**Proof** **5.** When the equilibrium point is (1, 0, 1), the Jacobian matrix is

$$\left[\begin{array}{ccc} {\varphi A\;-\;(\delta T+R}_{1}) & 0 & 0\\ 0 & {V+\delta T+U+\beta I+R}_{2}\;-\;C & 0\\ 0 & 0 & {P\;-\;V}_{c} \end{array}\right]$$

The eigenvalue $\lambda =\left[\begin{array}{c} {\varphi A\;-\;(\delta T+R}_{1})\\ {V+\delta T+U+\beta I+R}_{2}\;-\;C\\ {P\;-\;V}_{c} \end{array}\right]$ can be obtained by solving this point. If ${\varphi A\;<\;\delta T+R}_{1}$, ${C\;>\;V+\delta T+U+\beta I+R}_{2}$ and ${P\;<\;V}_{c}$ are established at the same time, then point (1, 0, 1) is the ESS of the system. If one or more eigenvalues are not negative, point (1, 0, 1) is a saddle point of the system.  □

**Explanation** **5.** *Under the equilibrium state of Proposition 5, the stable strategies of the government, enterprise, and the public are performance, non-R&D, and purchasing, respectively. In this case, both the government and the public have invested a certain cost in carbon reduction and emission reduction, while enterprises, as the main body of CPAN policy implementation, have chosen a non-R&D strategy. This is a worst-case scenario and not the problem we want to study*.

**Proposition** **6.** *If*${\varphi A\;<\;\delta T+R}_{1}$, ${V+\delta T+R}_{2}\;<\;C$*and*$V_{c}\;<\;P$*are established at the same time, then point (1, 0, 0) is the ESS of the system. Otherwise, point (1, 0, 0) is a saddle point of the system*.

**Proof** **6.** When the equilibrium point is (1, 0, 0), the Jacobian matrix is

$$\left[\begin{array}{ccc} {\varphi A\;-\;\delta T\;-\;R}_{1} & 0 & 0\\ 0 & {V+\delta T+R}_{2}\;-\;C & 0\\ 0 & 0 & V_{c}\;-\;P \end{array}\right]$$

The eigenvalue $\lambda =\left[\begin{array}{c} {\varphi A\;-\;\delta T\;-\;R}_{1}\\ {V+\delta T+R}_{2}\;-\;C\\ V_{c}\;-\;P \end{array}\right]$ can be obtained by solving this point. If ${\varphi A\;<\;\delta T+R}_{1}$, ${V+\delta T+R}_{2}\;<\;C$ and $V_{c}\;<\;P$ are established at the same time, then point (1, 0, 0) is the ESS of the system. If one or more eigenvalues are not negative, point (1, 0, 0) is a saddle point of the system.  □

**Figure 8:**
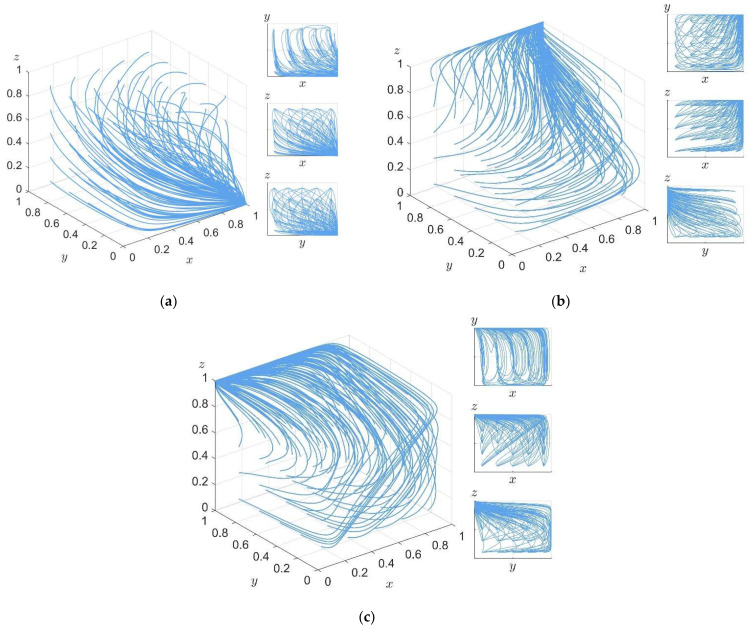
Computer simulation proof. (**a**) Proposition 6 system evolution 100 times results. (**b**) Proposition 7 system evolution 100 times results. (**c**) Proposition 8 system evolution 100 times results.

**Explanation** **6.** *In the equilibrium state of Proposition 6, the ESSs of the government, enterprise, and the public are performance, non-R&D and non-purchase, respectively. This situation generally occurs in the early implementation of CPAN policy and the carbon labeling system, when the government’s promotion of enterprises’ R&D of low-carbon technology and the public’s purchase of carbon label products have high benefits, while the R&D cost of enterprises’ low-carbon technology is high and the public’s willingness to purchase carbon label products is low. At this stage, due to their own short-sightedness, enterprises only focus on production interests rather than carbon emissions in the production process, so they generally choose a non-R&D strategy. Since the implementation of the carbon label system has just begun, the public’s lack of knowledge of carbon label products has led to a general choice of non-purchase strategies. Since CPAN policy is China’s strategic policy, the government should actively guide the development of low-carbon market at this time. Further, according to the relative size of each cost in Proposition 6, within the range required by Proposition 6, the simulation values A = 1, I = 2, T = 5, S = 2, R_1_ = 1, C = 10, V = 6, U = 2, P = 6, V_c_ = 3 are randomly set. The verification of Proposition 6 by computer simulation is shown in Figure 8a, and the verification results show that Proposition 6 is reliable*.

**Proposition** **7.** *If*${\theta S+\beta I+\varphi A\;<\;R}_{1}$, ${C\;<\;V+\delta T+U+\beta I+R}_{2}$*and*${P\;<\;\theta S+V}_{c}$*are established at the same time, then point (1, 1, 1) is the ESS of the system. Otherwise, point (1, 1, 1) is a saddle point of the system*.

**Proof** **7.** When the equilibrium point is (1, 1, 1), the Jacobian matrix is

$$\left[\begin{array}{ccc} {\theta S+\beta I+\varphi A\;-\;R}_{1} & 0 & 0\\ 0 & {C\;-\;(U+V+\delta T+\beta I+R}_{2}) & 0\\ 0 & 0 & {P\;-\;(\theta S+V}_{c}) \end{array}\right]$$

The eigenvalue $\lambda =\left[\begin{array}{c} {\theta S+\beta I+\varphi A\;-\;R}_{1}\\ {C\;-\;(U+V+\delta T+\beta I+R}_{2})\\ {P\;-\;(\theta S+V}_{c}) \end{array}\right]$ can be obtained by solving this point. If ${\theta S+\beta I+\varphi A\;<\;R}_{1}$, ${C\;<\;V+\delta T+U+\beta I+R}_{2}$ and ${P\;<\;\theta S+V}_{c}$ are established at the same time, then point (1, 1, 1) is the ESS of the system. If one or more eigenvalues are not negative, point (1, 1, 1) is a saddle point of the system.  □

**Explanation** **7.** *Under the equilibrium of Proposition 7, the stable strategies of the government, enterprise, and the public are performance, R&D, and purchase, respectively. This situation generally occurs in the implementation phase of the CPAN policy and carbon labeling system. Under the guidance of the government and the environmental crisis caused by excessive carbon emissions from enterprises, the public is dissatisfied with high-carbon enterprises and prefers low-carbon products. As the guide of CPAN policy, the government can intervene in the market of carbon label products and the low-carbon technology R&D of enterprises in this case, which can better improve public prestige and achieve considerable performance gains. For enterprises, due to the public demand for a low-carbon environment and the government’s good policy, they see the market value and potential value of carbon label products, so as to carry out low-carbon technology R&D. On the basis of proposition 6, according to the relative size of each cost in Proposition 7, the simulation values R_1_ = 3, V = 8, U = 5, R_2_ = 2, P = 5, and V_c_ = 6 are modified and randomly set in the range required by Proposition 7. Proof 7 is verified by computer simulation, as shown in Figure 8b. The verification results show that Proof 7 is reliable*.

**Proposition** **8.** *If*$R_{1}\;<\;\varphi A+\theta S+\beta I$, ${C\;<\;V+U+R}_{2}$*and*${P\;<\;V}_{c}$*are established at the same time, then point (0, 1, 1) is the ESS of the system. Otherwise, point (0, 1, 1) is a saddle point of the system*.

**Proof** **8.** When the equilibrium point is (0, 1, 1), the Jacobian matrix is

$$\left[\begin{array}{ccc} R_{1}\;-\;\left(\varphi A+\theta S+\beta I\right) & 0 & 0\\ 0 & {C\;-\;(V+U+R}_{2}) & 0\\ 0 & 0 & {P\;-\;V}_{c} \end{array}\right]$$

The eigenvalue $\lambda =\left[\begin{array}{c} R_{1}\;-\;\left(\varphi A+\theta S+\beta I\right)\\ {C\;-\;(V+U+R}_{2})\\ {P\;-\;V}_{c} \end{array}\right]$ can be obtained by solving this point. If $R_{1}\;<\;\varphi A+\theta S+\beta I$, ${C\;<\;V+U+R}_{2}$ and ${P\;<\;V}_{c}$ are established at the same time, then point (0, 1, 1) is the ESS of the system. If one or more eigenvalues are not negative, point (0, 1, 1) is a saddle point of the system.  □

**Explanation** **8.** *Under the equilibrium state of Proposition 8, the stable strategies of the government, enterprise, and the public are non-performance, R&D, and purchase, respectively. This situation generally occurs in the comprehensive implementation stage of CPAN policy and the carbon label system. When the carbon label product market has developed to a certain stage, the concept of low-carbon environmental protection is deeply rooted in the hearts of the people, and the guiding effect on enterprises and the public of the government’s policy investment decreases. At this stage, a mutually beneficial and symbiotic market environment for carbon label products is formed between enterprises and the public. Without government guidance, enterprises will still choose the low-carbon technology R&D strategy, and the public will also choose the carbon label product purchase strategy. At this time, on the basis of Proposition 7, according to the relative size of each cost in Proposition 8, within the range required by Proposition 8, the simulation values T = 1 and R_1_ = 1 are modified and randomly set. Proof 7 is verified by computer simulation, as shown in Figure 8c. The verification results show that Proof 8 is reliable*.

For the equilibrium point *(x, y, z)* in the solution domain Ω, it involves many parameters, and the Jacobian matrix is more complex. On the basis of the above Lyapunov indirect method, the experimental results in various environments can be obtained and analyzed by a computer system simulation method.

In summary, the final ESS of the three parties in the evolutionary game mainly depends on their respective costs and benefits, but the strength of different parameters may have an impact on the ESS state and convergence rate of the system, and the magnitude of this impact is difficult to explore through mathematical analysis. Therefore, in the next section, we explore the influence of the size of each parameter on the system evolution through a computer system simulation.

#### 3.3.3. System Simulation

On the basis of the above analysis, since China is currently in the early stage of CPAN policy implementation and the carbon label system has not been implemented nationwide, much real data in the model are difficult to obtain. Therefore, this paper used the simulation data to set the parameters of Proposition 6 as the initial state, that is, under the condition of satisfying the range of ${\theta S+\beta I+\varphi A\;<\;R}_{1}$*,* ${C\;<\;V+\delta T+U+\beta I+R}_{2}$*,* and ${P\;<\;\theta S+V}_{c}$; in the initial state, on the basis of the simulation values randomly set in Proposition 6, let *A = 1, I = 2, T = 5, S = 2, R1 = 1, C = 10, V = 6, U = 2, P = 6,* and *V_c_ = 3* in order to explore the influence of each parameter size on the evolution of the system.

**Figure 9:**
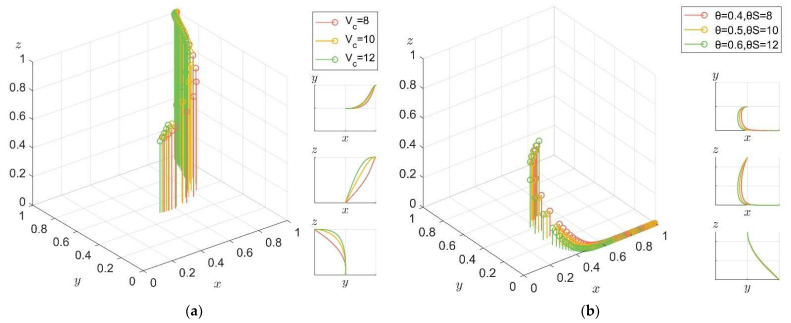
Evolution path of system under different product values and government subsidies. (**a**) Under different product values. (**b**) Under different government subsidies.

For the public, in order to explore the influence of different product values and government subsidies on their behavior of purchasing carbon label products from enterprises, first of all, the price *P* is kept unchanged and the product value is set as *V_c_ > P*, that is, *V_c_* is 8, 10, and 12. In order to exclude the interference of government policy factors, let *θS* = 0. Secondly, *θS* is set to 8, 10, and 12 to ensure the same thresholds. The amount of government subsidy is *S* = 20, and the execution intensity factor *θ* is set to 0.4, 0.5, and 0.6, representing the low, medium, and high execution intensity of the government, respectively. In order to eliminate the interference of product value factors, let *V_c_* = 0. The system evolution paths in the two cases are shown in Figure 9a and Figure 9b, respectively.

It can be seen from Figure 9a that when the product value is at a certain level, performance of the government, R&D of the enterprise, and purchase of the public are ESS for the system. Moreover, the higher the product value, the faster the public converges towards the “purchasing” direction. In addition, the choice of public strategy will also affect the choice of enterprise strategy. As can be seen from Figure 9b, in the case of the same government subsidies and product value, performance of the government, non-R&D of enterprise, and non-purchase of the public are the ESS for the system. Comparing Figure 9a,b, it can be seen that for the public, product value is more likely to encourage the public to choose the “purchasing” strategy than government subsidies at the same level. It can be seen that the product value of carbon label products has a greater impact on the public’s purchase intention than the government’s subsidy policy implementation. At the same time, the public’s demand for carbon label products has a positive effect on the enterprise’s choice of R&D strategies for low-carbon products.

**Figure 10:**
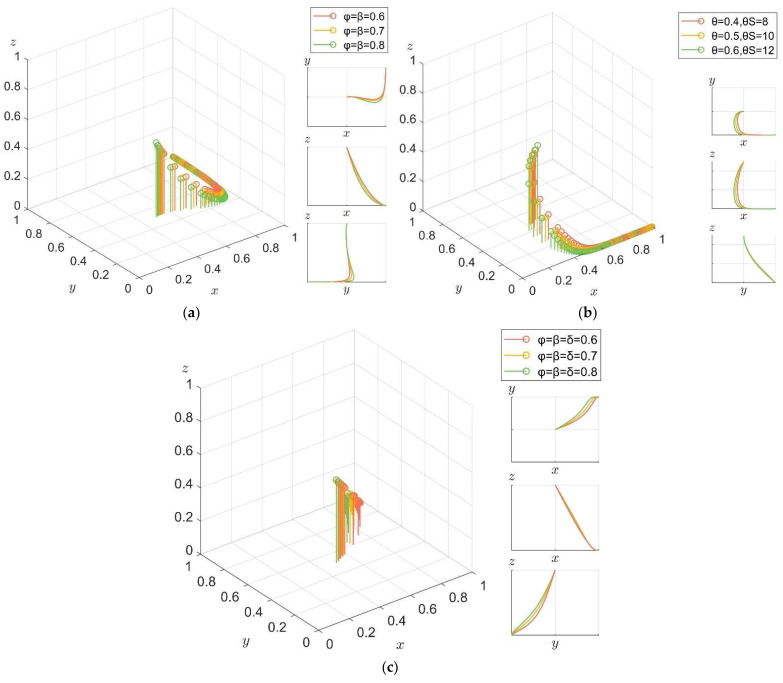
Impact of government guidance and punishment policy on low-carbon technology R&D behavior of enterprise. (**a**) Evolution path of the system under positive guidance policy. (**b**) Evolution path of the system under punishment policy. (**c**) Evolution path of the system under both policies.

For the enterprise, in order to explore the influence of the government’s positive guidance policy (including propaganda policy, incentive policy) and punishment policy on its low-carbon technology R&D behavior, with the original parameters unchanged, we set A+I=3 randomly, and set the execution intensity factors φ, β = 0.6, 0.7, 0.8. In order to exclude the influence of punishment policies on the experimental results, we set δ=0 to explore the influence of government only implementing positive guidance policies on enterprise behavior under high policy implementation intensity. The results are shown in Figure 10a. To ensure the same thresholds, we set T=3, δ=0.6, 0.7, 0.8. 

In order to exclude the influence of positive guidance policy on the experimental results, we set φ, β=0 to explore the influence of government only implementing punishment policy on enterprise behavior under high policy implementation intensity. The results are shown in Figure 10b. Finally, we set A+I=T=3, φ, β, δ=0.6, 0.7, 0.8 in order to explore the impact of the government’s positive guidance policy and punishment policy on enterprise behavior. The results are shown in Figure 10c.

It can be seen from Figure 6 that the government’s positive guidance policy and punishment policy have a promoting effect on the choice of the low-carbon technology R&D behavior of enterprise. Comparing Figure 10a,b, it can be seen that compared with the positive guidance policy, the punishment policy has a greater promoting effect on the choice of enterprise strategy. At the same time, Figure 10c shows that in real life, if the government can better combine positive guidance policy and punishment policy to guide the enterprise to carry out low-carbon technology R&D, the promotion effect is better than the single implementation of positive guidance policy or punishment policy.

**Figure 11:**
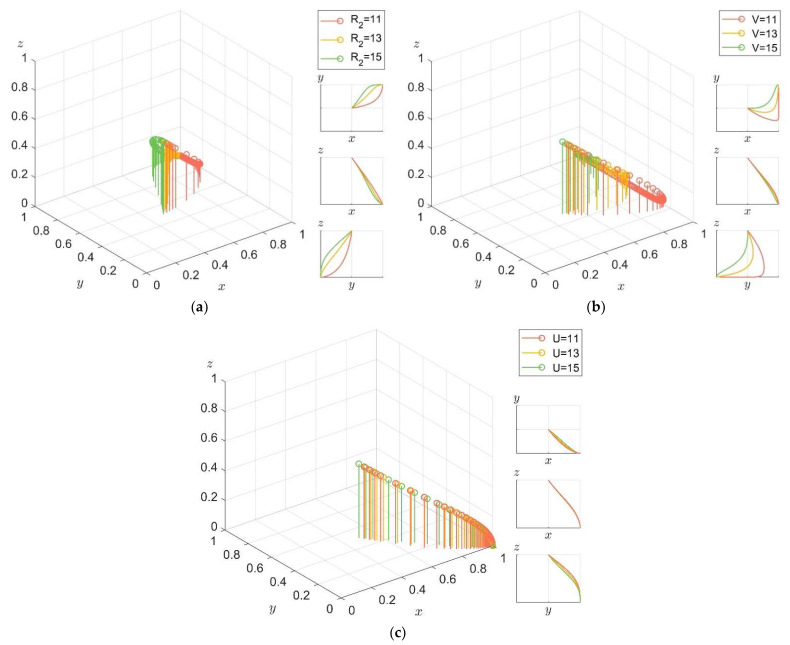
The impact of different income and opportunity costs on enterprise’s low-carbon technology R&D behavior and its evolution path. (**a**) Under different potential benefits. (**b**) Under different market returns. (**c**) Evolution path of the system under different opportunity costs.

For enterprises, in order to explore the influence of different income and opportunity costs on their choice of low-carbon technology R&D behavior, under the condition that the original parameters remain unchanged, we set *R_2_ > C* randomly as 11, 13, and 15. In order to exclude the interference of market value and opportunity cost of low-carbon products on the system, we set V=U=0 in order to explore the influence of public reputation and potential benefits on the enterprise. The results are shown in Figure 11a. Let *V > C*; to ensure the same thresholds, set V=11, 13, 15. In order to exclude the interference of potential market value and opportunity cost of low-carbon products on the system, let *R_2_ = U = 0* and explore the influence of market value of low-carbon products on enterprises when the R&D cost of low-carbon technology is relatively low. The results are shown in Figure 11b. Let *V > C*, in order to ensure the same thresholds, and set *U*=11, 13, 15. In order to exclude the potential market value of low-carbon products and the interference of market value on the system, let *R_2_ = V = 0* and explore the influence of opportunity cost on enterprises when the R&D cost of low-carbon technology is relatively low. The results are shown in Figure 11c.

It can be seen from Figure 11 that for different income thresholds, the higher the threshold, the faster the enterprise evolves towards low-carbon technology R&D. Under the same threshold, the positive effect of potential benefits on the enterprise’s choice of low-carbon technology R&D is more obvious, and the promoting effect of opportunity cost is smaller. Thus, for carbon label products, although various benefits can affect the behavior of enterprises, compared with the existing market value, enterprises pay more attention to the potential value of products, and the potential value is closely related to the public’s awareness of low-carbon environmental protection and carbon label product purchase intention.

## 4. Discussion

### 4.1. Implication

Through the method of evolutionary game, this paper innovatively explores the interaction and the micro-driving path between the policy guidance of the government, enterprises’ low-carbon technology R&D behavior and public purchase of carbon label products from the perspective of heterogeneity. Conclusions are listed below:

(1) In the “government-enterprise” evolutionary game model, the strategic choices of the government and enterprises are mainly affected by the relative size of their costs and benefits. However, the active performance of the government provides the possibility for enterprises to choose R&D strategies under low incomes. The effective guidance of the government is the key factor for enterprises to change short-sighted behaviors and carry out low-carbon technology R&D. And it is confirmed that strong public sentiment has a supervisory role in the choice of government accountability strategy and enterprises’ choice of low-carbon technology R&D strategy.
(2) In the “enterprise-public” evolutionary game model, the higher the product value is, the easier it is to attract the public to choose the carbon label product strategy. The higher the market value and potential benefits of carbon label products are, the easier it is to attract enterprises to choose R&D strategy. In addition, the model confirms that the effective government guidance can encourage both sides to choose strategies favorable to CPAN policy.
(3) The “government-enterprise-public” tripartite evolutionary game model is established on the basis of the first two models. The simulation results show that for the public, the product value of carbon label products has a greater impact on their purchase behavior than the government’s guidance and subsidy policies. For enterprises, compared with the government’s guidance policy, the government’s punishment policy has a greater role in promoting enterprises to choose low-carbon technology R&D strategy. Compared with a single policy, the implementation of positive guidance policy and punishment policy has a more obvious promoting effect on enterprises’ R&D of low-carbon products. In addition, potential earnings are more important than market returns and opportunity costs to promote the R&D behavior of low-carbon products.

### 4.2. Analysis of Different Models

On the basis of the mathematical deduction method used by Dong [39] and Xu’s tripartite evolutionary game model [30], this paper constructed the “government–enterprise–public” tripartite evolutionary game model. The superiority of this model is analyzed and demonstrated below. 

For the research on the relationship between government policies and low-carbon technology R&D, Dong mainly adopted the method of mathematical deduction. The research results showed that the impact of environmental regulation on low-carbon technology progress has a characteristic of “first suppressed and then increased”. This research expounds the influence of government behavior on enterprises low-carbon technology R&D behavior from a macro perspective. However, due to the limitation of methods, this model is difficult to utilize in terms of systematically explaining the micro-driving mechanism of enterprises’ low-carbon technology R&D behavior. On this basis, this paper used evolutionary game method to make up for this deficiency. In addition, the model also considers the role of public sentiment ignored in the above model and confirms that strong public sentiment has a supervisory effect on the government’ s choice of accountability strategies and enterprises’ choice of low-carbon technology R&D strategies.

Under the background of CPAN policy, Xu used the tripartite evolutionary game model to explain the interaction between the government, enterprise, and the public. The model introduces innovation technology incentive, pollution tax, public green environmental benefits, and other parameters, and explains the driving mechanism between the three players from the micro level. However, this study only focused on the regulatory role of the public on corporate low-carbon technology R&D, ignoring the consumer role of carbon label products played by the public in the market environment, and did not pay attention to the impact of market value and potential value of carbon label products and the opportunity cost of abandoning low-carbon technology R&D on enterprise strategy choice. On this basis, this study took the public as the consumer perspective of carbon label products; introduced the market value, potential value of carbon label products, the opportunity cost of abandoning low-carbon technology R&D, and other parameters; and supplemented the research results of Xu.

In summary, this paper used the tripartite evolutionary game method to better discuss the mutual driving mechanism of the government, enterprise, and the public under the background of CPAN policy from the micro level. At the same time, on the basis of Dong and Xu’ s research, this paper provides more comprehensive consideration to the parameter setting and used the Lyapunov indirect method to verify the stability and effectiveness of the model in different situations, and thus the constructed model is more universal.

## 5. Conclusions

Under the background of CPAN policy and the carbon labeling system, this paper innovatively researched from the perspective of heterogeneity. Firstly, the evolutionary game model of “government–enterprise” was constructed to explore the interaction between the two main participants under the carbon reduction policy. Secondly, this paper constructed a “enterprise–public” evolutionary game model to explore the interaction between the two important subjects in the carbon label market. Finally, on the basis of the first two models, the “government–enterprise–public” tripartite evolutionary game model was constructed, and the following conclusions and policy implications were drawn:

(1) Effective government regulation is the key factor in promoting low-carbon technology R&D. The central government can set up a low-carbon performance evaluation system to stimulate local government responsibility consciousness. In the context of the implementation of the CPAN policy and the carbon label system, local governments, as the implementers of the policy system, should adhere to the working principle of “saving first and two-wheel drive” proposed by the central government, effectively combining the positive guidance policy and punishment policy, encouraging and urging on enterprises to carry out low-carbon product R&D, increasing subsidies for enterprises’ carbon label products, and actively establishing the public’s consumption concept of low-carbon products so as to create a good atmosphere for low-carbon product R&D and a market environment for carbon label products.
(2) The value of carbon label products stimulates consumer buying more than government subsidies. In the R&D of low-carbon products, enterprises should pay more attention to product value research and development. A good market environment for carbon label products is complementary to the value of carbon label products provided by enterprises and the public’s low-carbon awareness. The ESS of the public mainly depends on the value of carbon label products provided by enterprises. High-quality carbon label products will bring about a broader market and more consumers to enterprises, and can also effectively reduce the cost pressure of low-carbon product R&D.
(3) Improving the public awareness of low-carbon products and helping enterprises to develop a low-carbon product R&D endogenous driving ability. The positive response of the public to the CPAN policy and the carbon label system is conducive to urging local governments to perform their responsibilities and enhancing corporate social responsibility. In addition, strong public sentiment will make enterprises pay attention to the potential market value of carbon label products. After the low-carbon products of enterprises enter into the market, the public’s purchase behavior will also increase the benefits of low-carbon product R&D for enterprises, which can guide enterprises’ low-carbon product R&D behavior into a benign endogenous evolution path.

Since China has not fully implemented the carbon label system under the background of CPAN policy, data on carbon label products such as market value and potential value of carbon label products are still unavailable. In the future work, this paper will supplement the model research data to further verify the effectiveness of the model.

## Figures and Tables

**Table 1:** Revenue matrix of “government–enterprise” parties considering public sentiment.

		Enterprise	
		R&D	Non-R&D
**Government**	**Performance**	$R_{1}{+R}_{g}\;-\;\varphi A\;-\;\beta I$ $,\;{V+R}_{2}+\beta I\;-\;C$	$\delta T+R_{1}-\varphi A-L_{g}$ ,−δT
	**Non-performance**	$R_{g}$ $,\;{V+R}_{2}-\;C$	$-L_{g}$ , 0

**Table 2:** The government-guided “enterprise–public” profit matrix.

		Enterprise	
		R&D	Non-R&D
**Public**	**Purchasing**	$V_{c}{+R}_{c}+\theta S\;-\;P$ $,{V+R}_{2}+\beta I\;-\;C$	$V_{c}{\;-\;P\;-\;L}_{c}$ ,−δT − U
	**Not purchasing**	$R_{c}$ $,{\beta I+R}_{2}\;-\;C$	${-L}_{c}$ ,−δT

**Table 3:** “Government–enterprise–public tripartite” income matrix.

		Enterprise			
		R&D		Non-R&D	
		Public Purchasing	Public Does Not Purchase	Public Purchasing	Public Does Not Purchase
**Government**	Performance	$R_{1}{+R}_{g}\;-\;\varphi A\;-\;\beta I\;-\theta S$ *,*	$R_{1}{+R}_{g}\;-\;\varphi A\;-\;\beta I,$	${\delta T+R}_{1}{\;-\;\varphi A\;-\;L}_{g}$ *,*	${\delta T+R}_{1}{\;-\;\varphi A\;-\;L}_{g},$
		${V+R}_{2}+\beta I\;-\;C$ *,*	$R_{2}+\beta I\;-\;C$ *,*	−δT − U *,*	−δT *,*
		$V_{c}{+R}_{c}+\theta S\;-\;P$	$R_{c}$	$V_{c}{\;-\;P\;-\;L}_{c}$	${-L}_{c}$
	Non-performance	$R_{g}$ *,*	$R_{g}$ *,*	${-L}_{g}$ *,*	${-L}_{g}$ *,*
		${V+R}_{2}\;-\;C$ *,*	$R_{2\;}-\;C$ *,*	−U *,*	0 *,*
		$V_{c}{+R}_{c}\;-\;P$	$R_{c}$	$V_{c}{\;-\;P\;-\;L}_{c}$	${-L}_{c}$

## Data Availability

The data used to support the findings of this study are available from the corresponding author upon request.
